# Advances in Research on Bladder Cancer Targeting Peptides: a Review

**DOI:** 10.1007/s12013-021-01019-3

**Published:** 2021-09-01

**Authors:** Bin Zheng, Pu Zhang, Heng Wang, Jinxue Wang, Zheng Hong Liu, DaHong Zhang

**Affiliations:** 1 Zhejiang Chinese Medical University, 310053 HangZhou, China; 2 Zhejiang Provincial People’s Hospital, Hangzhou Medical College, 310014 Hangzhou, China; 3 Handan Central hospital, 056001 Handan, China

**Keywords:** Bladder tumor, Peptides targeting, Diagnosis, Treatment, Research progress

## Abstract

Bladder cancer (Bca) is the second most common malignant tumor of the genitourinary system in Chinese male population with high potential of recurrence and progression. The overall prognosis has not been improved significantly for the past 30 years due to the lack of early theranostic technique. Currently the early theranostic technique for bladder cancer is mainly through the intravesical approach, but the clinical outcomes are poor due to the limited tumor-targeting efficiency. Therefore, the targeting peptides for bladder cancer provide possibility to advance intravesical theranostic technique. However, no systematic review has covered the wide use of the targeting peptides for intravesical theranostic techniques in bladder cancer. Herein, a summary of original researches introduces all aspects of the targeting peptides for bladder cancer, including the peptide screening, the targeting mechanism and its preclinical application.

## Introduction

Bladder cancer (Bca) is the second most common malignant tumor of the genitourinary system in Chinese male population with high potential of recurrence and progression up to 21–31% and 0.8–3.8%, respectively [1]. Currently a confirmed diagnosis of BCa mainly relies on cystoscopy or transurethral resection of bladder tumor combined with pathological examination [2]. On some occasion where non-muslce BCa (NMIBC) is of highly aggressive type, long-term intravesical is strongly recommended [2]. Therefore, at least 80% of BCa population benefit from the intravesical theranostic technique. However, both the surveillance of transurethral endoscope and intravesical therapy are lack of tumor specificity. Hence, carcinoma in situ or atypical hyperplasia can be ignored on the vision of white light cystoscopy, and drugs intravesically administrated can not be concentrated in the malignant lesion. The key to improve the efficacy of intravesical theranostic technique is to equip itself with targeting agent.

The currently used targeting agents can be mainly categorized into nucleic acid aptamers, antibodies, and peptides. Nucleic acid aptamers can be screened out to target many types of elements, including inorganic and organic small molecules, biological macromolecules, and even cells. But it is sometimes challenging to obtain nucleic acid aptamers with high specificity and high affinity for individual targets [3]. Due to the electronegative nature of the nucleic acid aptamers, the binding affinity for electronegative target wanes [4]. Its binding affinity also requires certain ionic strength, pH, and so forth [4]. The antibody exhibits good performance in good specificity, but its value is discounted by its obvious toxicity to normal tissues [5], poor penetration into tumor tissues [6, 7], and high cost of prodcution. The targeting peptide usually composes of no more than 100 monomeric amino acids. The cost of production is lower compared to that of antibodies. The high binding affinity can be maintained in harsh conditions. Besides, the lower molecular weight of the targeting peptide determined its facilitated elimination from nonspecific targeting tissues at the concentration of nanomolar. From the perspective of stability, specificity and economical cost, the targeting peptide is more potential to be used for modify the intravesical theranostic technique. However, no systematic review has covered the wide use of the targeting peptides for intravesical theranostic techniques in Bca. Herein, all aspects of the targeting peptides for Bca is introduced, including the peptide screening, the targeting mechanism, and its preclinical application.

## Screening of Peptide

### Phage-Displayed Peptide Libraries

One of the most widely used peptide screening technology is phage-displayed technology, featured by its high efficiency and low cost. Smith first proposed this technology in 1985 [8]. The mechanism behind this can be simplified as a combination of the genetic engineering technology and the peptide library with phage. In 1990, Scott and Smith successfuly introduced the fusion protein gIII containing random sequence peptides on the phage surface [9] to complete a phage surface display peptide library. Specifically, it is necessary to insert a chemically synthesized random oligonucleotide sequence into the gene encoding the phage coat protein to express various random short peptides Table 1. Then, the phages were panned for bladder tumor cells, and phages that specifically bind to bladder tumors were screened out. Amplify the DNA insert of the phage clone by polymerase chain reaction (Polymerase Chain Reaction, PCR), and sequence the DNA insert to convert it into the corresponding peptide sequence. The phage-displayed technology was used to screened out many types of BCa-targeting peptides, including P4 peptide, Bld-1 peptide, CLT1 peptide, and NYZL1 peptide [10–13].

**Table 1:** Summary of peptides

Name	Sequence	Screening method	Target	Advantages	Disadvantages	Applications	Reference
P4	NVFTVSP	Phage-displayed peptide libraries	High homology with the immunoglobulin-like (Ig-like) domain II–III (D2–D3) of the high-affinity FGF9 receptor (FGFR3c).	1. Increased the sensitivity of tumor cells to cisplatin 2. Therapeutic	Only has a higher affinity for tumors with abnormal FGFR expression	Therapeutic effect, which has certain tumor-specific cytotoxicity.	[10]
Bld-1	CSNRDARRC	Phage-displayed peptide libraries	Still not clear	Capable of distinguishing malignant cells from cells that exfoliate from benign tumors or inflammatory lesions as well as normal tissue.	The false-negative rate of urine exfoliated cell testing still needs to be reduced	Diagnostic function: it has the function of being able to target a specific tumor receptor	[11]
CLT1	CGLIIQKNEC	Phage-displayed peptide libraries	Depends on tumor cell integrin α5β1 and chloride intracellular channel 3 (CLIC3)	Strongly cytotoxic for tumor cells	Minimal cell death in tumor cells in the absence of plasma fibronectin	1. Diagnostic function: it has the function of being able to target a specific tumor receptor 2. Therapeutic effect, which has certain tumor-specific cytotoxicity.	[12]
NYZLl	cSSPIGRHc	Phage-displayed peptide libraries	Still not clear	Binding time more than 24 h	Low sensitivity	Appropriate for labeling with fluorophores for optical molecular cystoscopy diagnosis and treatment of NMIBC	[13]
PLZ4	cQDGRMGFc	One-bead one-compound combinatorial peptide library technology	Glycosylation or expression level of integrin may explain the preferential binding of PLZ4 to bladder cancer cell line over normal urothelial cells	Can be used for detection of subcutaneous tumor xenograft similar to metastatic bladder cancer	May reduce the degree of cell membrane penetration	May be used for imaging detection of bladder cancer and visualization of tumor location	[16, 44]
pHLIP	ACDDQNPWRAYLDLLFPTDTLLLDLLWA	Derived peptide	Tumor microenvironmental response (the formation of a helix across the lipid bilayer, triggered by the increase of peptide hydrophobicity due to the protonation of negatively charged residues induced by low pH.)	Generality and the absence of tumor heterogeneity issues.	May be difficult to distinguish between bladder inflammation and tumor	The peptide possesses dual delivery capabilities: it can tether cargo molecules to the cell surface and/or it can inject and release cell-impermeable cargo molecules into the cytoplasm	[28–30]
NT4	KPRRPYIL4	Derived peptide	Binds to neurotensin receptors	Conjugation to different functional units does not affect its binding properties	May lead to a reduction in cytotoxicity in vitro,	Conjugated with fluorescent probes can be used influorescence cystoscopy	[18, 45, 46]
R11	GRRRRRRRRRRR	Derived peptide	The high amount of phosphatidylserine and the overexpressed glycoprotein located on the outer surface of the BCa cell membrane might contributed to its targeting effiency.	1. Could get through a bladder surface lined with a sulphated polysaccharide glycosaminoglycan layer 2. Nuclear targeting	Has certain cytotoxicity	May be a delivery vector for agents used in treating bladder diseases.	[36, 42]

### One-Bead One-Compound Combinatorial Peptide Library Technology

The one-bead, one-peptide synthesis method is another peptide screening technology. One resin bead serves as a carrier, loading with a peptide of one sequence, and the One bead and one peptide (a resin bead contains a peptide sequence) are synthesized using resin as a carrier and according to the mixing-equalization method Table 2. After each step is connected to the resin, it is insoluble in the solvent, causing impurities. Excess reagents are easy to remove. After screening and sequencing of the obtained peptide library, the peptide structure can be obtained [14, 15]. The principle of the one-bead one-peptide library made by the mixing-equalization method is to first connect the protected amino acids to the resin, such as ten kinds of amino acids, after being mixed and deprotected, they are divided into ten groups, and each group has ten kinds connected to the resin. Then, ten protected amino acids are used to couple with each group to obtain 10 × 10 dipeptides (connected with resin), and the resulting dipeptides are mixed and divided into ten groups evenly. After repeating the above steps, 10X10X10 peptides can be obtained. Then use the established peptide library to develop tumor-specific targeting peptides. Simultaneously screen millions of beads (each bead is a unique peptide sequence) to identify peptides bound to tumor cell surface molecules. We can use an enzyme-linked colorimetric method similar to western blot or observe the cells attached to the beads’ surface to screen for “positive beads” with specific ligands. The PLZ4 peptide was obtained by co-cultivating the one-bead-one-peptide library with BCa cells after two-round screening, and has high affinity to BCa cells as proved in T24, 5637, and TCCSUP cell lines [16].

**Table 2:** The advantage and disadvantage of different targeting agents

Name	Advantages	Disavantages
Nucleic acid aptamers	1. Can be screened out to target many types of elements, including inorganic and organic small molecules biological macromolecules, and even cells 2. Easy to produce and cheap	1. Challenging to obtain nucleic acid aptamers with high specificity and high affinity for individual targets 2. Binding affinity for electronegative target wanes 3. Requires certain ionic strength, pH 4. Easily degraded
Antibodies	1. Good performance in good specificity	1. limited tissue penetration 2. high production cost
Peptides	1. Smaller in size and penetrate more efficiently into tissue compared to antibodies. 2. Synthesized by automated techniques with low production costs.	1. Have low stability in plasma, are sensitive to proteases and can be cleared from the circulation in a few minutes.

### Derived Peptide

The targeting peptide can also be a form of a natural ligand or a peptide derivative, such as pH low insertion peptide (pHLIP peptide). pHLIP is a water-soluble polypeptide derived from helix C of bacteriorhodopsin, and can insert into the cell membrane to form a stable transmembrane α-helix at the acidic tumor microenvironment [17]. Similarly, NT-4 peptide is a four-branched peptide containing human neurotensin sequence with short retention period in the circulation [18]. For prolonged circulation retention, several types of neurotensin analogs have been developed [19–21], and much more concentrated accumulation in BCa tissues was verified [18].

Fibronectin Attachment Protein (FAP) is a member of the fibronectin-binding glycoprotein family, and plays an essential role in the process of Bacillus Calmette-Guérin adhesion to BCa. The functional, active region of FAP is located in sequence 269–280, and the binding sequence is RWFV [22].

The purified HIV nuclear trans activating factor (TAT), a classical type of the cell-penetrating peptide (CPP), can be spontaneously internalized into the cell without a transfection agent in the cell medium [23]. The arginine-rich fragment in the 48–60 sequence of TaT protein is a crucial component to break through the membrane barrier independent of the receptor-mediated and the energy-dependent endocytosis pathway [24]. Arginine polymers composing of eleven arginines (R11) mimics the active part of the TAT peptide, and targets BCa specifically rather than other tumors [25].

## Peptide Targeting Mechanism

### Tumor Microenvironmental Response

Tumor cells are mainly metabolized by aerobic glycolysis to meet a strong energy demand, leading to the accumulation of the metabolic product lactic acid and the decreased pH value of the tumor tissue area being 0.3–0.7 U lower than normal tissues [26]. The pHLIP peptide is sensitive to targeting in an acidic environment and can detect changes in the body from 0.2–0.3 pH units [27, 28]. The acidic environment caused by the high metabolism of bladder tumors meets the conditions for specific targeting of PHLIP. For pHLIP, the reduced pH triggered the protonation of Asp residue and the increased hydrophobicity of the peptide chain. Under this condition, pHLIP can form its spiral structure easily across the bilayer membrane [29]. Studies on clinical specimens have confirmed the specific targeting of PHLIP to the acidic environment of bladder tumors [30].

### Receptor and Ligand Interaction

The cyclic peptide PLZ4 (amino acid sequence cQDGRMGFc) can binds to Bca cells specifically through the strong binding between the (D/N) GR motif and ανβ3 integrin [31]. When using U87 (human glioma cells) and MDA-231 (human breast cancer cells), both of which expresses high level of ανβ3 integrin [32], the amounts of PLZ4 internalized by U87 was higher than that in MDA-231. The results indicates more significant expression difference of glycosylation or integrin between normal urinary tract cells and BCa cells [10]. Furthermore, five amino acids, X2 (D), X4 (R), X7 (F), X3, and X6 (G) are proven to be critical amino acids for tumor-specific binding.

The P4 peptide sequence (NVFTVSP) shares the similar sequence with the conserved region of FGFR3c, and is expected to block the interaction between FGF9 and FGFR3c. The abnormal expression of FGF9 high-affinity receptor FGFR3c is closely related to the tumorigenesis and progression of BCa [33–35]. Thus, the P4 peptide is potential for inhibiting the downstream pathway of FGFR3c.

### Other Targeting Mechanisms

Among all CPPs, R11 is the best candidate for BCa-targeting regardless of the cell variety [36]. But the mechanism remains to be explored. The high amount of phosphatidylserine and the overexpressed glycoprotein located on the outer surface of the BCa cell membrane might contributed to its targeting effiency.

The CLT1 peptide gains the ability to target the BCa in the presence of a urothelial tumor matrix component fibronectin, and then the cellular internalization can be driven through the α5β1 integrin and chloride intracellular channel 3 (CLIC3). For the purpose of CLT1 internalization, the origin of fibronectin appears to be irrelevant, as both plasma fibronectin, cellular fibronectin and urine fibronectin are able to work [37].

Neurotensin-quadruple neurotensin peptide (NT4) is a derivative of Neurotensin, and its branched structure protects it from the proteolysis and brings enhanced biological activity [38]. In vitro cell lines and tissue specimen proved the BCa-targeting property of NT4, but whether the neurotensin receptor is the real binding site remains unknown, as the neurotensin receptor coexist under the urothelial and in the smooth muscle layer.

## Application of Peptides

### The Diagnostic Effect of Peptides on Bladder Tumors

Combining the BCa-targeting peptide with fluorescein or imaging agents enable more precise visualization of BCa in different modalities.

pHLIP conjugated with a near-infrared fluorescent dye [indocyanine green (ICG)] targets low extracellular pH, allowing visualization of malignant lesions in human bladder carcinoma ex vivo. Cystectomy specimens obtained after radical surgery were irrigated with a solution of the ICG pHLIP construct, and the fluorescence spot image was collected to establish the correlation between ICG pHLIP imaging and histopathological analysis. The detection sensitivity is 97%. The specificity is 100%, but reduced to 80% when transurethral resections or chemotherapy is applied prior to the CG pHLIP imaging [39].

R11, as an unique BCa-targeting CPP, is used to functionalize the superparamagnetic iron oxide particles (SPIO) to improve the MR imaging contrast. Results from Prussian blue staining and TEM revealed that SPIO-R11 were efficiently taken up by BCa cells rather than normal urothelial cells, and in vitro MRI studies showed that SPIO-R11 greatly increased MR imaging contrast over the SPIO in BCa cells [25].

PLZ4 (amino acid sequence: cQDGRMGFc) was identified that could selectively bind to Bca cell lines and three primary Bca cells from human patients, but not to normal urothelial cells, cell mixtures from normal bladder specimens, fibroblasts, and blood cells [16]. After verifying the non-targeting properties of FITC, it can be connected to PLZ4 to detect cancer cells that shed urine.

### Application in Treatment

The CLT1 peptide selected from the phage peptide library is a tumor homing peptide related to the clotted plasma of tumor stroma [40]. An in-depth understanding of peptides found that it can induce unfolded protein response and autophagy cell death in proliferating endothelial cells, thereby possessing antiangiogenic activity in vivo [41]. Also, the cytotoxicity of fibronectin and CLT1 peptide is significantly increased after forming a complex. The P4 peptide, with its antitumor properties, also screened through the phage peptide library, binds to FGF9 to counteract the aggressive phenotype induced by FGF9, including cell migration, invasion, and proliferation in vitro and inhibition of tumor growth in vivo. In research, FGF9 reduces the function of cisplatin-induced apoptosis to enhance drug resistance, and the antagonism of P4 to FGF9 can effectively enhance drug sensitivity [42].

Previous studies have shown that the peptide at the C-terminus of p53 (p53C) can restore the binding and transactivation functions of the mutated p53-specific DNA sequence and cause p53-related tumor cell apoptosis [43]. p53C itself cannot enter tumor cells spontaneously to play a role. Incorporating p53C specifically into tumor cells is the direction of our research. Nonpolar and large macromolecules cannot pass through the cell membrane, but R11 can transport hydrophilic substances and macromolecular proteins. R11 binds to p53C to deliver p53C to Bca cells. The results fully prove that R11 (R11-p53C) binding peptide can effectively target in situ and metastatic bladder tumor cells and inhibit tumor cell growth [42]. The R11-p53C peptide is a potential drug for bladder urothelial cancer treatment, whether for in situ or metastatic bladder tumors.

Liposome polyethylene glycol can enhance the stability of particles in the bladder cavity and prevent particle aggregation, but how to solve low retention in the treatment of bladder tumors remains to be explored. Teams have developed targeted preparations, such as micellar nanocarriers surface-modified PLZ4 ligand and liposome-linked RWFV peptides to form peptide biopolymers. The advantage of these carriers is that they can encapsulate different therapeutic drugs and imaging agents. And it can be targeted to bladder tumor tissue, which can be used for tumor imaging and flexible replacement of drugs [43].

In addition to coupling with nanocarriers that encapsulate tumor chemotherapy drugs, peptides can also be directly coupled to antitumor drugs. The three lysine cores of NT4 provide other linking groups that can be used to connect other functional units [39]. Under the endoscope, the NT4 peptide is combined with a fluorescent group first, and the tumor is labeled and then combined with the chemotherapy drug. A simple “exchange” of functional units can achieve individualized treatment. Whether it is through nanocarriers or directly coupled with antitumor drugs, the tumor localization function of targeting peptides is used to internalize antitumor drugs into target tumor tissues, which not only improves the level of intracellular medications but also reduces systemic side effects.

## Conclusions and Future Perspectives

The targeted polypeptide is an amino acid sequence that binds explicitly to different targets. It has become a hot research topic in recent decades and has a wide range of applications in biomedicine. Compared with the existing urine exfoliated cell line examination, the targeting peptide for Bca has higher sensitivity. The combined use with cystoscope significantly improves the tumor detection rate of the cystoscope. Targeting peptides have good target tissue positioning and internalization capabilities. After being mixed with imaging agents, it can effectively detect tumors that are not easy to find in the early stage, and after replacing the treatment ligand, it plays the role of targeted therapy, reducing the systemic side effects of the drug. Although targeted peptides have many advantages, they also face some problems. For example, how to maintain the stability of peptide molecules in the body and control the rate of degradation. Although polypeptides made from D-amino acids are more stable than other structure amino acids, they are not suitable for specific receptor binding applications due to the change in chirality. With the deepening of research, targeted peptides are expected to be used in the early diagnosis of Bca, targeted therapy, and follow-up examinations, and provide new methods for diagnosing and treating Bca.

In summary, most types of targeting peptides were screened out by involving specific types of cells as models, thus lead to Their targeting approaches noneffective in covering some aspects of tumor-targeting mechanisms, that is to say, these peptides might be that nonfunctional when exposed to tumor heterogeneity. Behaviors of targeting peptides and their complexes with cargos were different. The size and physiochemical properties of cargos might interfere with the targeting pathway. Current researches focus little on the role of targeting peptides in tumor promoter or inhibitor, exposing the unpredictable risk. Although the current targeting peptides still have shortcomings, their infinite potential deserves more attention. Once these targeted peptides are approved for clinical use, they will be of great help to the diagnosis, treatment, and follow-up examination of Bca.
